# Rapid Quantification of Melamine in Different Brands/Types of Milk Powders Using Standard Addition Net Analyte Signal and Near-Infrared Spectroscopy

**DOI:** 10.1155/2016/9256102

**Published:** 2016-07-20

**Authors:** Bang-Cheng Tang, Chen-Bo Cai, Wei Shi, Lu Xu

**Affiliations:** ^1^Institute of Applied Chemistry, College of Material and Chemical Engineering, Tongren University, Tongren, Guizhou 554300, China; ^2^College of Chemistry and Life Science, Chuxiong Normal University, Chuxiong 675000, China; ^3^The Modernization Engineering Technology Research Center of Ethnic Minority Medicine of Hubei Province, College of Pharmacy, South-Central University for Nationalities, Wuhan 430074, China

## Abstract

Multivariate calibration (MVC) and near-infrared (NIR) spectroscopy have demonstrated potential for rapid analysis of melamine in various dairy products. However, the practical application of ordinary MVC can be largely restricted because the prediction of a new sample from an uncalibrated batch would be subject to a significant bias due to matrix effect. In this study, the feasibility of using NIR spectroscopy and the standard addition (SA) net analyte signal (NAS) method (SANAS) for rapid quantification of melamine in different brands/types of milk powders was investigated. In SANAS, the NAS vector of melamine in an unknown sample as well as in a series of samples added with melamine standards was calculated and then the Euclidean norms of series standards were used to build a straightforward univariate regression model. The analysis results of 10 different brands/types of milk powders with melamine levels 0~0.12% (w/w) indicate that SANAS obtained accurate results with the root mean squared error of prediction (RMSEP) values ranging from 0.0012 to 0.0029. An additional advantage of NAS is to visualize and control the possible unwanted variations during standard addition. The proposed method will provide a practically useful tool for rapid and nondestructive quantification of melamine in different brands/types of milk powders.

## 1. Introduction

Dairy products are essential components of a healthy diet for human and are very popular in all age groups owing to their high nutritional value and pleasurable flavor [1–3]. Recent years have seen greatly increasing production and consumption of dairy products in the world [4, 5]. An important factor that influences the textures and flavors of dairy products is the protein content, which has been adopted as a quality index by many industries. Unfortunately, because the traditional Kjeldahl method for analysis of total nitrogen content as an indication of protein levels is insufficient to distinguish between organic nitrogen and protein and nonprotein sources, unethical manufacturers deliberately added some illegal exogenous materials to dairy products to obtain an incorrectly higher readout of apparent protein content [6–8].

One of the most notorious exogenous adulterants used in dairy products is melamine, chemically known as 2,4,6-triamino-1,3,5-triazine [9–11]. Melamine is a nitrogen-rich (about 66.6%) heterocyclic triazine produced on a large scale (1.2 million tonnes in 2007) [12, 13]. It is primarily used in the synthesis of melamine formaldehyde resins for the production of paper finishers, flame retardant, commercial filters, moulding compounds, wrinkle-free textile, and many other materials [14–16]. As a very cheap and readily available industrial material, melamine was added to various food and food-related products, including milk, infant formula, frozen yogurt, biscuits, candy, coffee drinks, pet food, and feed [17–19], to increase the nitrogen level and to reduce costs. Although melamine has low toxicity by itself, when combined with cyanuric acid and uric acid, it can form insoluble crystals, which may lead to kidney stones, eventual renal failure, and ultimately death [20]. The most severe outbreak of melamine contamination occurred in 2008 in China, which had caused kidney stones in thousands of people and at least six deaths of young children [21].

To prevent further contamination and frauds, maximum limits have been established for melamine in infant formula and other foods by many countries. The emergent need for regulation of melamine has promoted extensive and intensive laboratory efforts to develop rapid, widely available, and cost-effective methods for analysis of melamine in various samples [1, 12, 18, 22–24], including capillary electrophoresis [25], high-performance liquid chromatography (HPLC) [26, 27], LC with mass spectrometry (LC-MS) [28, 29], gas chromatography with MS (GC-MS) [30, 31], micellar LC [32, 33] matrix-assisted laser desorption/ionization MS (MALDI-MS) [34], nuclear magnetic resonance spectroscopy [35], vibrational spectroscopy [1] and imaging [36, 37], chemiluminescence analysis [38, 39], electrochemical analysis [40], and immunoassay [41]. Among various methods, near-infrared (NIR) spectroscopy, as a rapid and nondestructive analytical method widely used in food analysis [42, 43], is promising for high-throughput screening and detection of melamine [44]. Although NIR has a comparatively lower sensitivity and higher detection limit compared with other methods, it was demonstrated to be sufficient in detecting excessive use of melamine in dairy products and could provide a convenient tool to rapidly screen and quantify melamine in Chinese markets [17, 45]. For quantitative analysis, chemometric methods for multivariate calibration (MVC), such as partial least squares (PLS), support vector machines (SVM), and artificial neural network (ANN), have also been successfully used to calibrate melamine levels in adulterated samples [1].

Although MVC combined with NIR spectroscopy has shown good accuracy and precision in analysis of melamine for some specific samples, the practical application of ordinary MVC to different brands/types of samples can be largely limited because the prediction of a new sample from an uncalibrated group would be subject to a significant bias due to matrix effect. This problem can be solved by performing calibration transfer [46] or standard addition [47]. While calibration transfer is a complicated procedure with high requirements of the practitioners' expertise and its performance can be influenced by many factors, standard addition method is a relatively easy-to-use tool for analysis of samples with complex compositions and from diverse origins.

Net analyte signal (NAS) theory [48] has been proven to be useful in the development of new multivariate calibration methods, evaluation of the figures of merit of multivariate calibration, variable selection, outlier diagnosis, and data preprocessing. NAS is based on an intuitive idea, namely, separating a part of the signal that is directly related to the concentration of the analyte of interest from that of interfering components. Using the Euclidean norm of the computed NAS vector, the multivariate signal can be represented as a univariate scalar. In this way, the multivariate calibration can be reduced to a simple univariate linear regression, which is especially useful for model validation and prediction. A multivariate standard addition method using NAS (SANAS) [49, 50] has been suggested and demonstrated to be effective in overcoming the matrix effect or indirect interferences.

The objective of this work was to study the feasibility of using multivariate standard addition method and NIR spectroscopy for rapid quantification of melamine in milk powders of different brands/types. To control the possible variations in preparation and measurement of added samples, the SANAS method was used to analyze different brands/types of milk powder samples.

## 2. Materials and Methods

### 2.1. Preparation of Melamine-Adulterated Milk Powder Samples and Standard Addition

Ten different brands/types of milk powder samples were collected from the quality inspection departments of producers as shown in Table 1. The shelf lives of all the samples were equal or over six months and, to the date of analysis, no sample has expired. The milk powder samples were kept in a dark, cool, and dry area at about 25°C with complete packaging before preparation and analysis. A melamine (Sinopharm Chemical Reagent Co., Ltd., Shanghai, China) gradient consisting of 5 levels, namely, 0.01, 0.02, 0.04, 0.08, and 0.12 percent (w/w%), together with the unadulterated samples, was prepared for each of the 10 batches. Therefore, 60 adulterated milk powder samples were obtained and used for analysis by SANAS. For standard addition, a designed gradient of 4 melamine standards was added to each level of the above 60 samples as listed in Table 2.

### 2.2. NIR Spectral Measurements

The NIR diffuse reflectance spectra of milk powder samples were measured in the spectral range from 4000 to 10000 cm^−1^ on an Antaris II Fourier transform-NIR spectrometer (Thermo Electron Co., Waltham, Massachusetts, USA) using the RESTLT 3.0 software. All samples were measured with a PbS detector and an internal gold background as the reference. The resolution was 8 cm^−1^ and the scanning interval was 3.857 cm^−1^. Therefore, each spectrum had 1557 individual data points for chemometric analysis. For each object, 32 scans were performed and more scans did not enhance the spectral signals significantly.

### 2.3. Computation of Net Analyte Signal (NAS) Vector

For a detailed description of the net analyte signal (NAS) theory, one can refer to [48]. In this work, only a brief introduction is presented. For bilinear spectral data like NIR, the NAS of a chemical component is defined as the part of its pure spectrum that is orthogonal to the spectral space spanned by all the interfering components. The NAS vector of the *k*th component in a multicomponent mixture is defined as

(1) $$\begin{array}{c} NAS_{k}=\left(\;I-R_{-k}R_{-k}^{+}\;\right)s_{k}, \end{array}$$

where **R**_−*k*_ is a matrix whose columns contain the pure spectrum of each component in the mixture except the *k*th component; **s**_*k*_ is the pure spectrum of the *k*th component; and **I** is a unit matrix and the superscript “+” represents the Moore-Penrose pseudo inverse of a matrix. The spectral variations caused by instrumental and environmental disturbances are also included in **R**_−*k*_. Using the data matrix of calibration mixtures reconstructed by principal components analysis (PCA) or partial least squares (PLS), a rank annihilation procedure is adopted to compute **R**_−*k*_:

(2) $$\begin{array}{c} R_{-k}=R_{rec}-\alpha r{\hat{c}}_{k}^{T}, \end{array}$$

where **R**_rec_ is the PCA- or PLS-reconstructed data matrix of interfering components using *A* significant components; ${\hat{c}}_{k}$ is the analyte concentration vector **c**_*k*_ explained by the *A* significant components; and **r** is a vector including the spectrum of the *k*th analyte. Although **r** can be the pure spectrum of analyte *k*, **s**_*k*_, or the spectrum of a mixture including analyte *k*, the pure spectrum **s**_*k*_ is the best choice because it contains maximal information on the *k*th analyte. *α* is a scalar factor which can be computed as

(3) $$\begin{array}{c} \alpha =\frac{1}{{\hat{c}}_{k}^{T}R_{rec}^{+}r}. \end{array}$$

### 2.4. Standard Addition Method Using NAS

To do standard addition, a set of *n* standard solutions of the *k*th analyte were added to each of the samples. The concentration data of standards were collected in the vector **c**_*s*_. The NIR data of the serial spiked samples were collected in the matrix, **R**_sa_ (*n* × *p*), where *p* is the number of NIR channels. The spectrum of a sample without standard addition is collected in a column vector, **r**_un_. By subtracting **r**_un_ from each column of **R**_sa_, one can obtain a matrix, **R**_*s*_ (*n* × *p*), which contains the spectra of the standards in the presence of the matrix effect. **R**_*s*_ is subject to PCA and the reconstructed **R**_*s*_ is used to rebuild **R**_sa_ and **R**_sa,rec_ is obtained, and then the matrix of interfering components, **R**_−*k*_, can be readily computed according to (2) and (3).

Subsequently, the NAS vectors of the *k*th analyte for the standard addition samples can be computed as

(4) $$\begin{array}{c} NAS_{sa,k}=\left(\;I-R_{-k}R_{-k}^{+}\;\right)R_{sa,rec}. \end{array}$$

By plotting the Euclidean norm of the row vectors of NAS_sa,*k*_ (*n* × *p*) against **c**_*s*_, the standard addition curve as for the univariate SA can be obtained to derive the concentration of *k*th analyte in the unknown sample.

### 2.5. Software

All the data analysis was performed using MATLAB 7.10.0 (R2010a) platform (MathWorks, USA). The data preprocessing and SANAS algorithms were performed based on in-house computational coded scripts written by authors in MATLAB.

## 3. Results and Discussion

**Figure 1:**
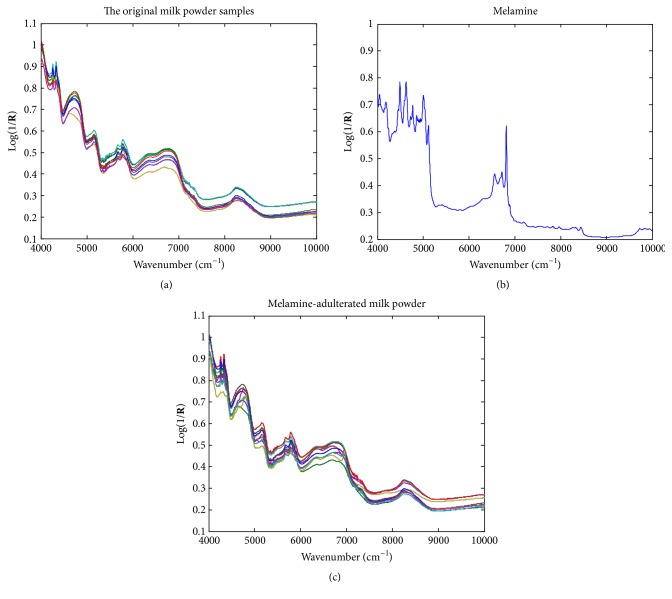
The NIR spectra of the original milk powder samples, pure melamine, and adulterated milk powder samples.

**Figure 2:**
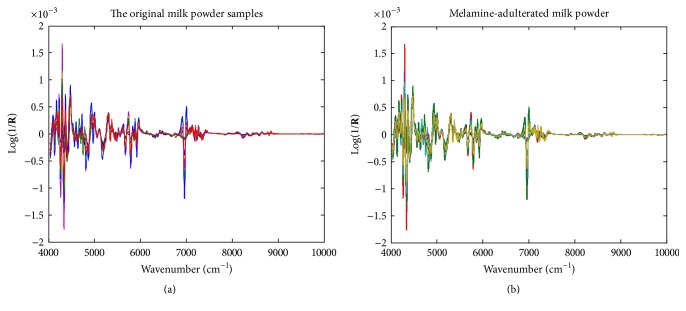
The second-order derivative (D2) spectra of the original and melamine-adulterated milk powder samples.

The NIR spectra of the original milk powder samples and pure melamine as well as the melamine-adulterated samples are demonstrated in Figure 1. For ease of peak attribution, chemical bonds are denoted as atom–atom, where an atom can be carbon (C), hydrogen (H), oxygen (O), and nitrogen (N). For the spectra of milk powder, the peak around 4258 cm^−1^ is the combination absorbance of C–H symmetric stretching and C–H bending, and those at 4335 cm^−1^ can be attributed to the combination absorbance of C–H antisymmetric stretching and C–H bending. Other peak assignments are as follows: 4750 cm^−1^, combination of the basebands of N–H stretching and bending; 5157 cm^−1^, combination of the basebands of O–H stretching and bending; ~5700 cm^−1^, the first overtones of C–H stretching in various groups; ~6500 cm^−1^, the first overtone of N–H stretching; ~6900 cm^−1^, the first overtone of O–H stretching; and ~8300 cm^−1^, the second overtones of C–H stretching in various groups. By comparing the spectra of milk powder and melamine, the most significant difference is the intensive peak of melamine at 6811 cm^−1^, which could be attributed to the baseband of N–H antisymmetric stretching.  Figure 2 demonstrates the second-order derivative (D2) spectra of the original and melamine-adulterated milk powder samples. However, because the added concentrations of melamine were very low (0.01~0.12%, w/w), no significant changes were found from the spectra of the adulterated milk powder by the naked eye. Multivariate calibration models were developed on the raw and D2 spectra using SANAS.

**Figure 3:**
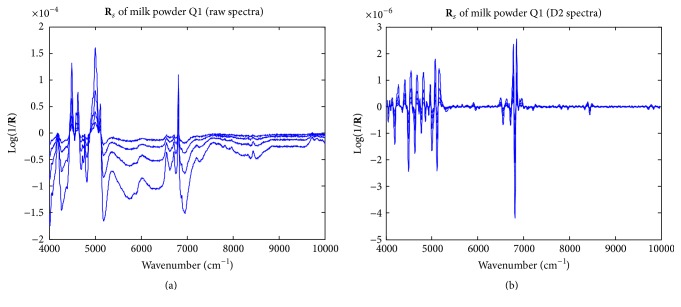
The **R**_*s*_ matrix computed for milk powder sample Q1.

**Figure 4:**
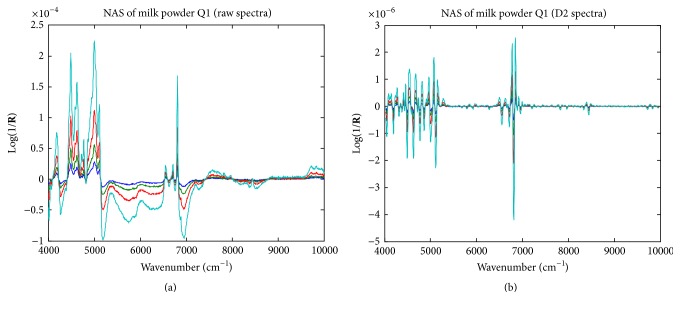
The NAS vectors of milk powder sample Q1.

For each unknown sample, in order to compute the NAS vector, the spectrum of the original sample was subtracted from each of the melamine-spiked samples to obtain the matrix **R**_*s*_. The resultant spectra in **R**_*s*_ for the sample Q1 in Table 1 are plotted in Figure 3. Seen in Figure 3, the high similarity between the raw spectra in **R**_*s*_ and that of pure melamine indicates that the computation of **R**_*s*_ using the method proposed in SANAS was very effective and helpful for accurate estimation of the NAS vectors. To rebuild **R**_*s*_, **R**_sa,rec_ was obtained using the primary principal components (PCs) of **R**_*s*_. Because there were only 4 columns in **R**_*s*_ and its data structure was simple, the number of primary PCs was estimated according to an intuitive criterion that 95 percent of the total variances of **R**_*s*_ should be explained. The NAS vectors of the 4 standard-added samples were computed according to (2)–(4).  Figure 4 demonstrates the computed NAS vectors of the 4 standard addition samples for the milk powder Q1.

Theoretically, the melamine level in an unknown sample could be estimated by plotting the Euclidean norm of the computed NAS vectors, ‖NAS_sa,*k*_‖, against the concentrations of added standards, **c**_*s*_. However, because the number (60) of unknown samples to be analyzed was large, least squares regression (LSR) was performed between ‖NAS_sa,*k*_‖ and **c**_*s*_:

(5) $$\begin{array}{c} NAS_{sa,k}=ac_{s}+b. \end{array}$$

**Figure 5:**
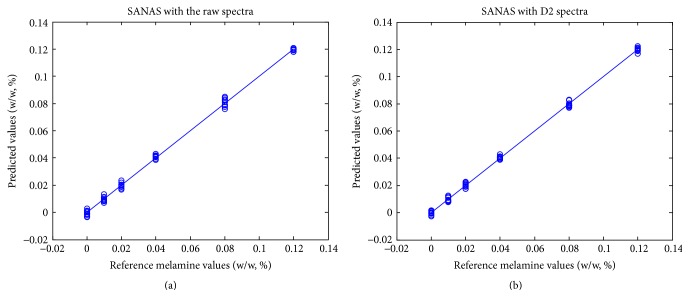
The prediction results and reference values of melamine levels by SANAS.

By setting the value of NAS_sa,*k*_ to be zero, the melamine level of the unknown samples can be computed as *c*_unknown_ = *b*/*a*. For all the 60 unknown samples, the correlation coefficients (*r* ^2^) of the above LSR were over 0.953, indicating that the computation of NAS vectors was very accurate. The prediction results of the 60 melamine-adulterated milk powder samples are summarized in Table 3. Seen in Table 3, the root mean squared error of prediction (RMSEP) of melamine ranged from 0.0012 to 0.0029 with different levels of melamine to be analyzed. The prediction accuracy of SANAS was not significantly influenced by the content of melamine. Moreover, the prediction performance by SANAS using the raw spectra and D2 spectra was similar although the results with D2 spectra seemed to be slightly better than those by the raw spectra, indicating that the unwanted spectral variations caused by baseline shifts were well controlled during standard addition. The predicted melamine levels were plotted against the reference values as shown in Figure 5, also indicating that the prediction errors were low and approximately uniform for different melamine levels to be analyzed.

To further evaluate the figures of merit of the method, the selectivity, sensitivity in terms of limit of detection (LOD), linearity (Pearson's *r* ^2^), and the accuracy and precision in terms of mean relative standard deviation (RSD) were computed and listed in Table 4. In China and the US, the maximum residue levels (MRL) for infant formula are 1.0 mg/kg and 2.5 mg/kg for milk and other dairy products, respectively [51]. Although the LOD (0.0025%) of this method was much worse than the regulation standards, this method provides the potential of rapid analysis and screening of the frauds in Chinese markets, where the practical melamine contents were much higher (up to 2563 mg/kg) [52].

## 4. Conclusions

The feasibility of using NIR and SA for rapid quantification of melamine in different brands/types of milk powders was investigated. The analysis results for the 10 batches of melamine-adulterated milk powder samples demonstrate that SANAS is an effective method for SA multivariate calibration, which can visualize and control the spectral variations caused during SA in univariate regression. Moreover, the calibration accuracy was not significantly influenced by melamine levels to be analyzed. Compared with traditional multivariate calibration, combination of NIR and SANAS will provide a more practically applicable method for analysis of melamine in different brands/types of milk powder without requiring complex calibration transfer procedures.

## Figures and Tables

**Table 1:** The original milk powder samples of 10 different batches.

Number	Codes of brands/types	Types^a^	Production date
1	Q1	Skimmed, regular	May 3, 2015
2	Y1	Semiskimmed, high-calcium	Apr. 13, 2015
3	Q2	Skimmed, regular	May 22, 2015
4	M1	Semiskimmed, regular	Jun. 7, 2015
5	A1	Whole, sweet	May 19, 2015
6	Y2	Whole, sweet, high-calcium	Apr. 15, 2015
7	M2	Whole, high-calcium	Jun. 7, 2015
8	Y3	Skimmed, regular	May 12, 2015
9	M3	Skimmed, high-calcium	Jun. 9, 2015
10	Q3	Whole, regular	Apr. 27, 2015

^a^The term “regular” means no deliberate addition of calcium or other elements to the milk; the term “high-calcium” denotes addition of calcium to the samples by the producers; the term “sweet” denotes addition of sucrose.

**Table 2:** Composition of serial samples spiked with different amounts of melamine standard.

Sample levels	Melamine content (%, w/w)	Gradient of melamine standard added (%, w/w)
1	0	0.01, 0.02, 0.04, and 0.08
2	0.01	0.01, 0.03, 0.07, and 0.11
3	0.02	0.02, 0.06, 0.10, and 0.14
4	0.04	0.04, 0.08, 0.12, and 0.20
5	0.08	0.04, 0.08, 0.16, and 0.24
6	0.12	0.04, 0.12, 0.20, and 0.36

**Table 3:** The prediction results of melamine levels in 10 different batches of milk powder samples by SANAS using the raw and D2 spectra.

Melamine level (w/w, %)	Raw spectra		D2 spectra	
	RSD^a^ (%)	RMSEP^b^	RSD (%)	RMSEP
0	—	0.0019	—	0.0014
0.01	19.5	0.0019	17.3	0.0016
0.02	9.8	0.0019	9.2	0.0017
0.04	4.8	0.0018	3.1	0.0012
0.08	3.8	0.0029	2.6	0.0020
0.12	1.3	0.0014	1.2	0.0014

^a^RSD: relative standard deviation.

^b^RMSEP: root mean squared error of prediction.

**Table 4:** The figures of merit of melamine analysis in 10 different batches of milk powder samples by D2 spectra.

Selectivity	Sensitivity (LOD^b^, w/w%)	Linearity (*r* ^2^)	Accuracy (RSD^a^)	Precision (RSD)
0.614	0.0025	0.953	5.80%	3.74%

^a^RSD: relative standard deviation.

^b^LOD: limit of detection.
